# The streptococcal phase-variable type I restriction modification system SsuCC20p dictates the methylome of *Streptococcus suis* impacting the transcriptome and virulence in a zebrafish larvae infection model

**DOI:** 10.1128/mbio.02259-23

**Published:** 2023-12-08

**Authors:** Thomas J. Roodsant, Boas van der Putten, Jaime Brizuela, Jordy P. M. Coolen, Tim J. H. Baltussen, Kim Schipper, Yvonne Pannekoek, Kees C. H. van der Ark, Constance Schultsz

**Affiliations:** 1 Department of Global Health, Amsterdam Institute for Global Health and Development, Amsterdam UMC, University of Amsterdam, Amsterdam, the Netherlands; 2 Department of Medical Microbiology and Infection Prevention, Amsterdam UMC, University of Amsterdam, Amsterdam, the Netherlands; 3 Department of Medical Microbiology, Radboud University Medical Centre, Nijmegen, the Netherlands; Institut Pasteur, Paris, France; University of Bologna, Bologna, Italy

**Keywords:** *Streptococcus suis*, zoonosis, type I restriction modification system, phase-variation, DNA methylation, epigenetic regulation, transcriptome

## Abstract

Phase-variable type I restriction modification (RM) systems are epigenetic regulatory systems that have been identified in numerous human bacterial pathogens. We previously showed that an emerging zoonotic lineage of *Streptococcus suis* acquired a phase-variable type I RM system, named SsuCC20p. The SsuCC20p locus was identified in the genome of multiple streptococcal species, indicating that it is not restricted to *S. suis* and can be acquired through horizontal gene transfer. We demonstrate that SsuCC20p phase variability relies on a recombinase present within the locus. SsuCC20p is the single RM system responsible for the genome methylation profiles that were detected in the representative zoonotic *S. suis* isolate 861160 *in vitro*. In addition, we show that, contrary to previous observations, *hsdS* genes located downstream of *hsdM* and the recombinase gene contribute to the SsuCC20p genome methylation profile. SsuCC20p locked mutants expressing a single *hsdS* each show a unique genome methylation profile and, when grown in human serum, have distinct transcriptomes. In a zebrafish larvae infection model, we observed significant differences in virulence between the locked mutants and a corresponding shift in *hsdS* allele distribution in the wild type. These data indicate that the streptococcal phase-variable type I RM system SsuCC20p can impact bacterial fitness via epigenetic regulation of gene expression, which impacts the virulence of *S. suis* in the zebrafish larvae infection model.

**IMPORTANCE:**

Phase variation allows a single strain to produce phenotypic diverse subpopulations. Phase-variable restriction modification (RM) systems are systems that allow for such phase variation via epigenetic regulation of gene expression levels. The phase-variable RM system SsuCC20p was found in multiple streptococcal species and was acquired by an emerging zoonotic lineage of *Streptococcus suis*. We show that the phase variability of SsuCC20p is dependent on a recombinase encoded within the SsuCC20p locus. We characterized the genome methylation profiles of the different phases of SsuCC20p and demonstrated the consequential impact on the transcriptome and virulence in a zebrafish infection model. Acquiring mobile genetic elements containing epigenetic regulatory systems, like phase-variable RM systems, enables bacterial pathogens to produce diverse phenotypic subpopulations that are better adapted to specific (host) environments encountered during infection.

## INTRODUCTION

*Streptococcus suis* is an opportunistic bacterial pathogen in pigs and an emerging zoonotic pathogen (1). Human infections can lead to meningitis, streptococcal toxic shock-like syndrome, and septicemia (2, 3). Human infections are linked to exposure to pigs, such as (occupational) handling of pig (products) or consuming undercooked or raw pig products (2, 4). *S. suis* is classified into serotypes based on capsular polysaccharides (CPS) structure and into sequence types, which in turn are clustered into clonal complexes, based on its genomic background as assessed by multi-locus sequence typing. Most human infections are caused by *S. suis* serotype 2 of clonal complex (CC) 1, although infections with other serotypes (e.g., serotype 14) and genotypes (e.g., CC20) have also been reported (3, 5).

In the Netherlands, a unique zoonotic serotype 2 CC20 clade has been identified, which is more closely related to the non-zoonotic but virulent serotype 9 CC16 clade than to the zoonotic serotype 2 CC1 clade (5). Three major genomic differences in the accessory genome between CC16 and CC20 have been postulated to contribute to zoonotic potential of CC20 strains (5). These include a capsule switch through acquisition of a serotype 2 CPS locus (i); acquisition of an 89k pathogenicity island, previously identified in Chinese zoonotic outbreak isolates (ii); and acquisition of an 18.5-kb prophage region with a complete type I restriction modification (RM) system with phase-variable specificity subunits named SsuCC20p (iii) (5, 6).

**Fig 1:**
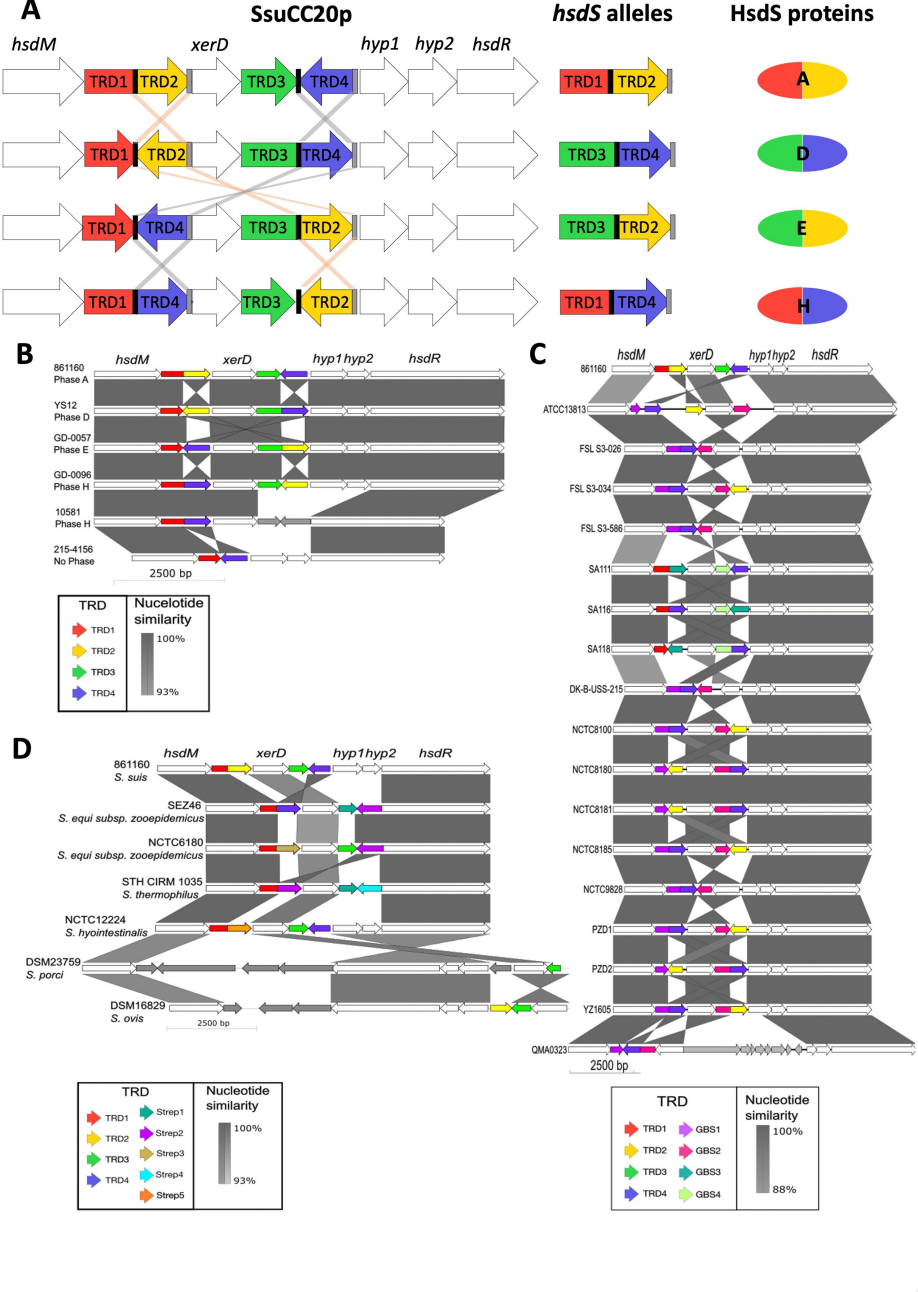
SsuCC20p presence in *S. suis* and other streptococci. (A) Graphical representation of SsuCC20p. The black bars represent two identical left inverted repeats (TAAATCATCATTTA), and the gray bars (TAAATGATGATTTA) represent two identical right inverted repeats. Target recognition domains (TRDs) are numbered 1 to 4. Gray and orange lines indicate observed recombination events. (B) Visualization of unique tblastn hits for SsuCC20p presence in *S. suis*, (C) *S. agalactiae*, or (D) other streptococcal species genomes visualized with easyfig. Unique TRDs that are not found in *S. suis* are named GBS1–4 for TRDs found in *S. agalactiae* and Strep1–5 for TRDs found in other streptococcal species.

Phase-variable RM systems can be found in many pathogenic bacteria and have been shown to regulate bacterial virulence (7). Type I RM systems consist of three host specificity determinants (*hsd*) genes encoding a specificity subunit (S), a modification subunit (M), and a restriction subunit (R) (Fig. 1A) (8). The subunits can form a pentameric complex (HsdS, 2HsdM, and 2HsdR) with endonuclease activity and a trimeric complex (HsdS and 2HsdM) with methylase activity (8). HsdS is a DNA-binding protein that determines the target DNA sequences of both complexes. HsdS consists of two separate target recognition domains (TRDs) that each recognize a specific part of the target DNA sequence. The TRDs in the HsdS protein are spatially separated from each other, which in turn separates the TRD-recognized DNA motifs by multiple nucleotides (8). Phase-variable type I RM systems have multiple TRD regions that can recombine to form different functional HsdS proteins. TRD recombination is mediated via inverted repeats (IRs), which in some systems is (partially) mediated by a recombinase within the same locus (8–10). Unique *hsdS* alleles, when expressed with an *hsdM*, give unique methylation profiles in the genome. The methylation of the genome can affect gene expression by affecting the binding of regulatory proteins, such as transcription factors, to regulatory sequences upstream of genes (11, 12). In this way, the phase-dependent methylation profiles can impact virulence, as was shown for *Streptococcus pneumoniae* (13).

We characterized SsuCC20p and showed that SsuCC20p is phase variable, expressed, actively methylates the *S. suis* genome and affects the transcriptome. In a zebrafish larvae infection model for bacterial infection (14–23), isogenic locked mutants with distinct genome methylation profiles showed differences in virulence.

## RESULTS

### SsuCC20p is present in multiple streptococcal species

The SsuCC20p locus contains an *hsdM*, an *hsdR*, four TRDs of which two form a functional *hsdS*, a site-specific recombinase gene (*xerD*), and two hypothetical proteins (Fig. 1A). TRD2 and TRD4 are both flanked by an identical set of IRs. Since the discovery of SsuCC20p, many additional *S. suis* genomes have been sequenced (5, 6). In 1,749 *S*. *suis* genome assemblies, we searched for the presence of the SsuCC20p locus (Fig. 1A; Table S1). A total of 22 isolates carried the complete SsuCC20p locus. Two isolates (215-4156 and 216-4157) missed *xerD*, TRD2, and TRD3 and had lower identity (78% and 79%) with hypothetical protein 2. One isolate (10581) missed both hypothetical proteins, TRD2 and TRD3 (Fig. 1B; Table S2). Additional strains in which SsuCC20p was identified include two human isolates from Germany (1) and the Czech Republic (1) and four pig isolates from Australia (2), the US (1), and Denmark (1). Besides CC20 isolates, SsuCC20p was found in one strain belonging to CC25 (1) and in three strains not yet assigned to a CC. Four different *hsdS* alleles were found in the assemblies of *S. suis* genomes, which encoded four unique HsdS proteins (Fig. 1A and B). Allele H was found most frequently within CC20 genomes (*n* = 10), followed by A (*n* = 6) and E (*n* = 5). Allele D was only found within one Chinese ST17 isolate. All except one isolate were associated with disease (Table S2). All genome assemblies with a complete SsuCC20p locus contained a single complete *hsdS*. No strains were found to carry two complete *hsdS* or only truncated *hsdS*. In addition, a potential single recombination event between the IR upstream of *xerD* and at the end of TRD3, leading to an inverted orientation of the *xerD*, was not observed in any of the *S. suis* genome assemblies.

SsuCC20p is located on a prophage region and is therefore likely acquired via horizontal gene transfer (6). To identify additional species that carry the same type I RM system, we searched the NCBI Refseq Genomes Bacterial Database for the presence of each individual gene within the SsuCC20p locus. SsuCC20p was restricted to streptococci, and most hits (17/23) were found in *Streptococcus agalactiae. S. agalactiae* isolates contained some TRDs identical to SsuCC20p but also contained unique TRDs named GBS1–4 (Fig. 1C; Table S3). All but one *S*. *agalactiae* strain had a functional *hsdS* comprised of two TRDs, and five strains had three instead of four TRDs within the locus. In these five *S*. *agalactiae* strains, the orientation of *xerD* was inverted compared to the rest of the locus, and these strains had lost the IRs identified in *S. suis* SsuCC20p. SsuCC20p was also identified in five other streptococcal species but with more genomic differences than found for the *S. agalactiae* isolates, including loss of genes, acquisition of a new TRDs, or large intergenic insertions (Fig. 1D; Table S3).

### SsuCC20p is phase variable

**Fig 2:**
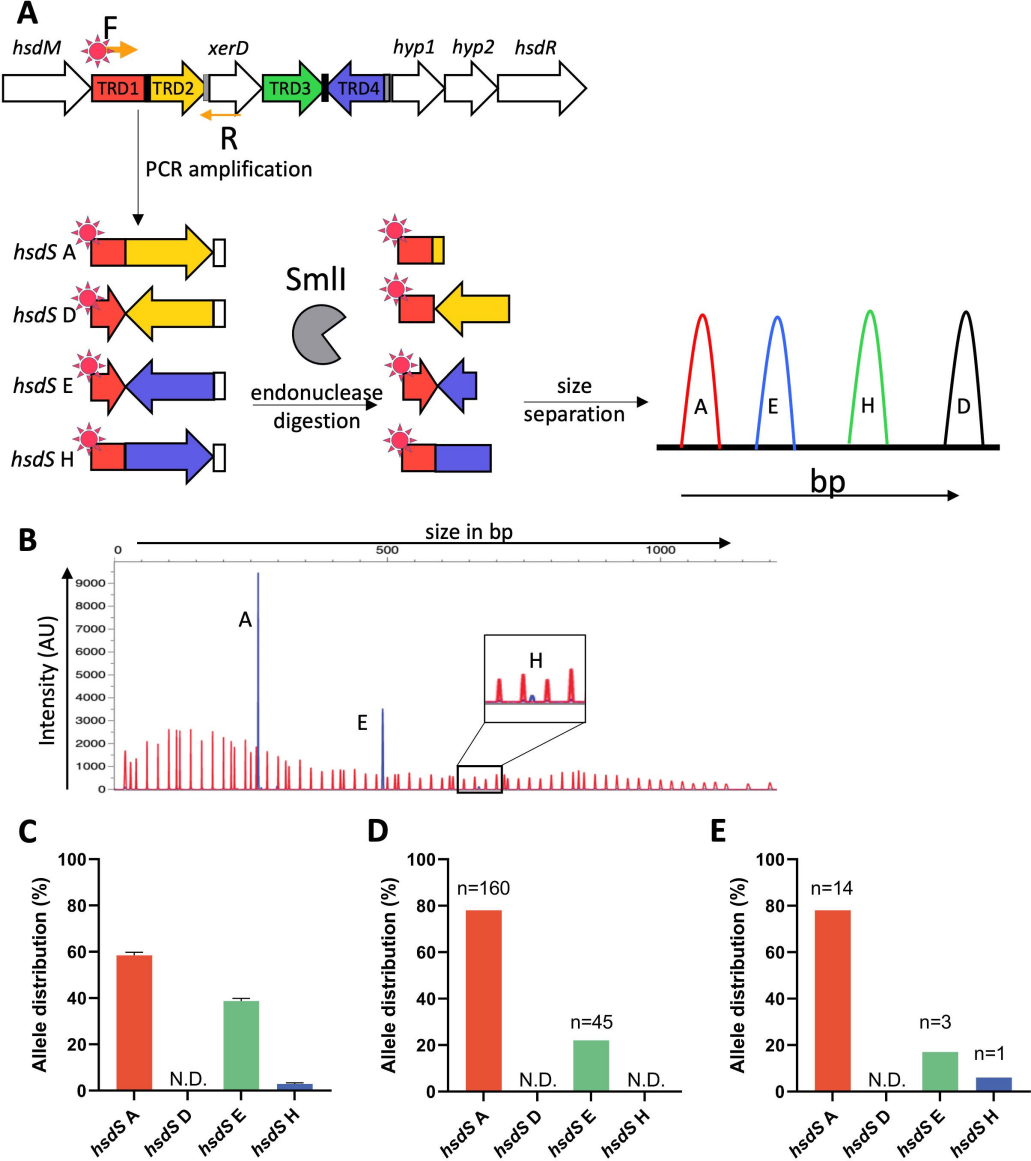
SsuCC20p is phase variable, and three alleles can be found within a single isolate. (A) Graphical representation of the hsdS allele quantification by FAM-labeled PCR product endonuclease digestion and fragment analysis. (B) Representative example of WT 861160 hsdS allele identification; red peaks are the LIZ1200 size marker, and blue peaks, the FAM-labeled fragments. (C) hsdS allele distribution was computed by measuring the relative area under the curve of the different hsdS alleles using PeakScanner v3.0 in three biological replicates. (D) hsdS alleles were quantified using PacBio or (E) Oxford Nanopore long-read sequencing data; read count per allele is indicated above each bar. N.D., not detected; AU, arbitrary units.

The presence of a site-specific recombinase (*xerD*) and TRDs flanked by IRs within a single locus, combined with the presence of multiple *hsdS* alleles in different genome assemblies, suggests that SsuCC20p is phase variable. While four different alleles of SsuCC20p have been identified in CC20 *S. suis* genomes, the presence of these different alleles within a single isolate has not been demonstrated yet. We chose the zoonotic ST20 isolate 861160 as our model strain because a single contig assembly of its genome is available (24). We identified and quantified the allelic variants after overnight growth in Todd-Hewitt broth with 0.5% yeast extract (THY) in a FAM-labeled PCR with a subsequent endonuclease digestion and fragment analysis (Fig. 2A), as previously used to quantify phase-variable *hsdS* alleles (9, 13, 25). The most prevalent allelic variant was *hsdS* A, in accordance with the *hsdS* allele present in the 861160 genome assembly (GCA_902702745), followed by *hsdS* E and *hsdS* H (Fig. 2B and C). Allele D was undetected, in line with its absence in the assembled genomes of ST20 isolates. Using long-read sequencing, we corroborated this allele distribution within strain 861160 (Fig. 2D and E). Again, none of these reads harbored multiple complete *hsdS* alleles, only truncated *hsdS* or an inverted *xerD*, which was confirmed by PCR (Fig. S1).

### SsuCC20p is expressed and differentially methylates the genome

**Fig 3:**
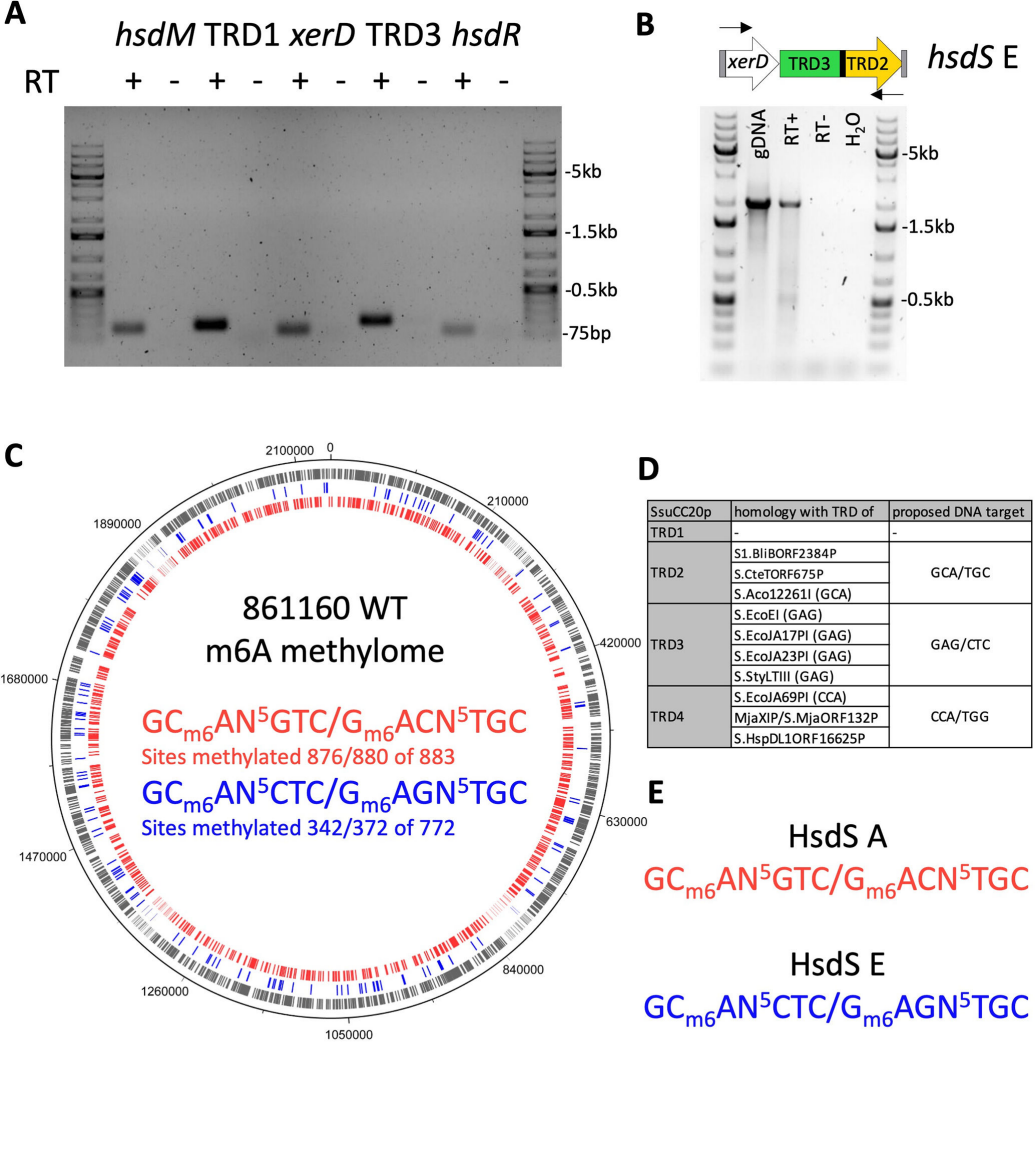
hsdS alleles downstream and upstream of xerD are expressed and methylate the genome. (A) Expression of SsuCC20p genes was verified by PCR on reverse-transcribed RNA, using gene-specific qPCR primers. PCR products were run on a 1% agarose gel. RT + or − indicates the presence of reverse transcriptase in the reverse transcription reaction; marker lanes were loaded with the 1 kb+ ladder. (B) PCR amplification of xerD-hsdS E fragment from genomic DNA (gDNA) and cDNA. RT + or − indicates the presence of reverse transcriptase in the reverse transcription reaction; marker lanes were loaded with the 1 kb+ ladder. (C) 861160 WT methylome; m6A methylated sites in genome are indicated on the genome with the color corresponding to the DNA motif; gray indicates sites that have either of the two DNA motif sites. (D) Homology of SsuCC20p TRDs to other type I RM system hsdS genes; identified DNA target sequences are indicated between brackets; for details, see Fig. S2. (E) Proposed target DNA motif of HsdS A and HsdS E.

In previously identified type I RM systems, the *hsdS* allele that is not located directly downstream of the *hsdM* gene is silent because it is encoded on the opposite strand, lacks a start codon, or lacks a promoter (26). In SsuCC20p, a complete and functional *hsdS* is predicted not only directly downstream of the *hsdM* gene in alleles A and H but also downstream of *xerD* in alleles D and E (Fig. 1A). To investigate if both TRD1 (alleles A and H) and TRD3 (alleles D and E), as well as the other genes of SsuCC20p, are transcribed in 861160, we performed RT-PCR. All genes of SsuCC20p were expressed in 861160 when grown into logarithmic phase in THY (Fig. 3A). Additionally, we confirmed that *hsdS* E (TRD3-TRD2) is transcribed while downstream of *xerD* by RT-PCR (Fig. 3B).

To determine if SsuCC20p methylates the genome, the methylome of wild-type 861160 grown in THY was compared with a Δ*hsdS* strain lacking each of the four TRDs and *xerD* using PacBio HiFi sequencing. In the wild type (WT), two m6A methylation profiles were identified with the type I RM system characteristic bipartite buildup of the target DNA motifs (8), and both methylation profiles were absent in the Δ*hsdS* strain (Fig. 3C). Almost all potential sites of GCAN^5^GTC/GACN^5^TGC (99%/100%) within the genome were methylated, but the GCAN^5^CTC/GAGN^5^TGC sites were only partially methylated (44%/48%). This partial methylation (37%/30%) was also observed in a second PacBio HiFi sequencing run on newly isolated genomic DNA (Fig. S2). The methylated sites had overlap between both runs, but each run also had unique m6A methylations sites (Fig. S2).

To assign the *hsdS* alleles found in WT 861160 (A, E, and H) to the two identified methylation profiles, we searched for homologues of the *hsdS* alleles for which the DNA targets were identified, using InterPro (v5.55-88.0, default settings)(27), which gave hits for TRD2, TRD3, and TRD4 (Fig. S3). Based on the protein identity, we predict that TRD2, TRD3, and TRD4 recognize GCA/TGC, GAG/CTC, and CCA/TGG, respectively (Fig. 3D). In 861160 WT, *hsdS* A and *hsdS* E are the dominant alleles and responsible for the observed methylation profiles. Both *hsdS* alleles contain TRD2, which likely recognizes GCA/TGC (Fig. 3D). *hsdS* E also has TRD3, which likely recognizes GAG/CTC (Fig. 3D). Based on the detected InterPro hits, the observed methylation profiles, and dominant *hsdS* alleles, we propose that GC_m6_AN^5^GTC/G _m6_ACN^5^TGC is methylated by HsdS A and GC_m6_AN^5^CTC/G_m6_AGN^5^TGC by HsdS E (Fig. 3E).

### Phase variability of SsuCC20p is *xerD* dependent

**Fig 4:**
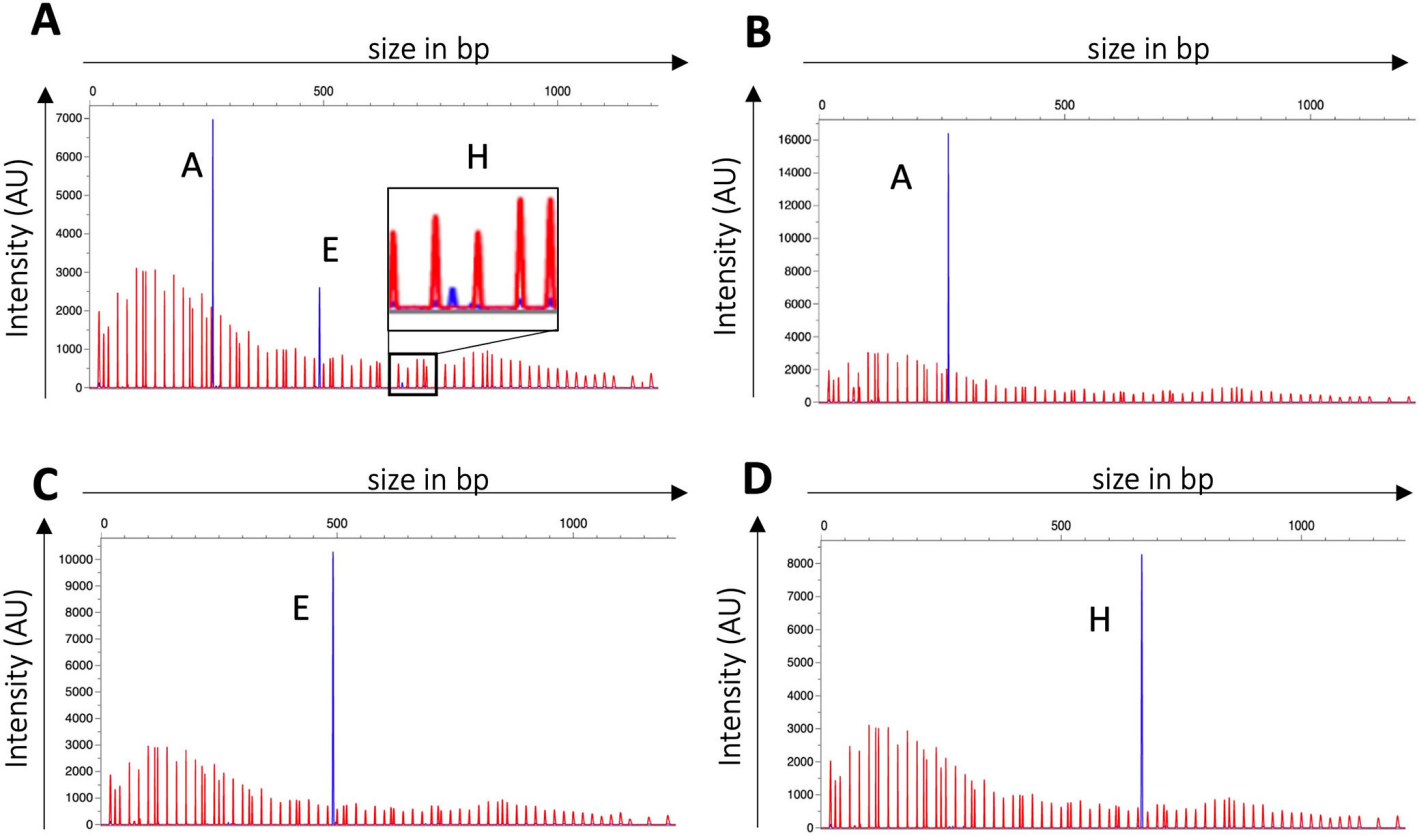
SsuCC20p phase variability is xerD dependent. (A) Representative example of WT (A) and AxerD (B–D) hsdS allele identification after subculturing in THY broth. Red peaks are the LIZ1200 size marker, and blue peaks, the FAM-labelled fragments; hsdS allele identification was analyzed with PeakScanner v3.0 in three biological replicates. AxerD mutants showed a single hsdS allele, which was hsdS A, E, or H depending on the hsdS allele present in the genome upon mutating xerD.

In *S. pneumoniae,* the site-specific recombinase (*creX*) present in the type I RM system SpnIII is essential for the TRD recombination between the small IRs (15 bp), but not for TRD recombination between the larger IRs (85 bp and 333 bp) (9). Given that the IRs within SsuCC20p are 14 bp, we expected that TRD recombination in SsuCC20p is mediated by the *xerD* present in the locus, which was tested in Δ*xerD* mutants in 861160. After three sequential subcultures, to allow for recombination, the *hsdS* alleles present in the Δ*xerD* mutants and WT strain were assessed. In the WT, all three *hsdS* alleles were detected (Fig. 4A), but in the Δ*xerD* mutants, only a single *hsdS* allele per mutant was identified, which was *hsdS* A, E, or H depending on the *hsdS* allele present in the genome upon mutating *xerD* (Fig. 4B through D). Long-read sequencing confirmed the fixed state of the *hsdS* alleles in the Δ*xerD* mutants. In addition, SMRT and HiFi sequencing showed that the kanamycin cassette used to create the mutants disrupted SsuCC20p genome methylation (Table S4).

### *hsdS* alleles give unique methylomes

**Fig 5:**
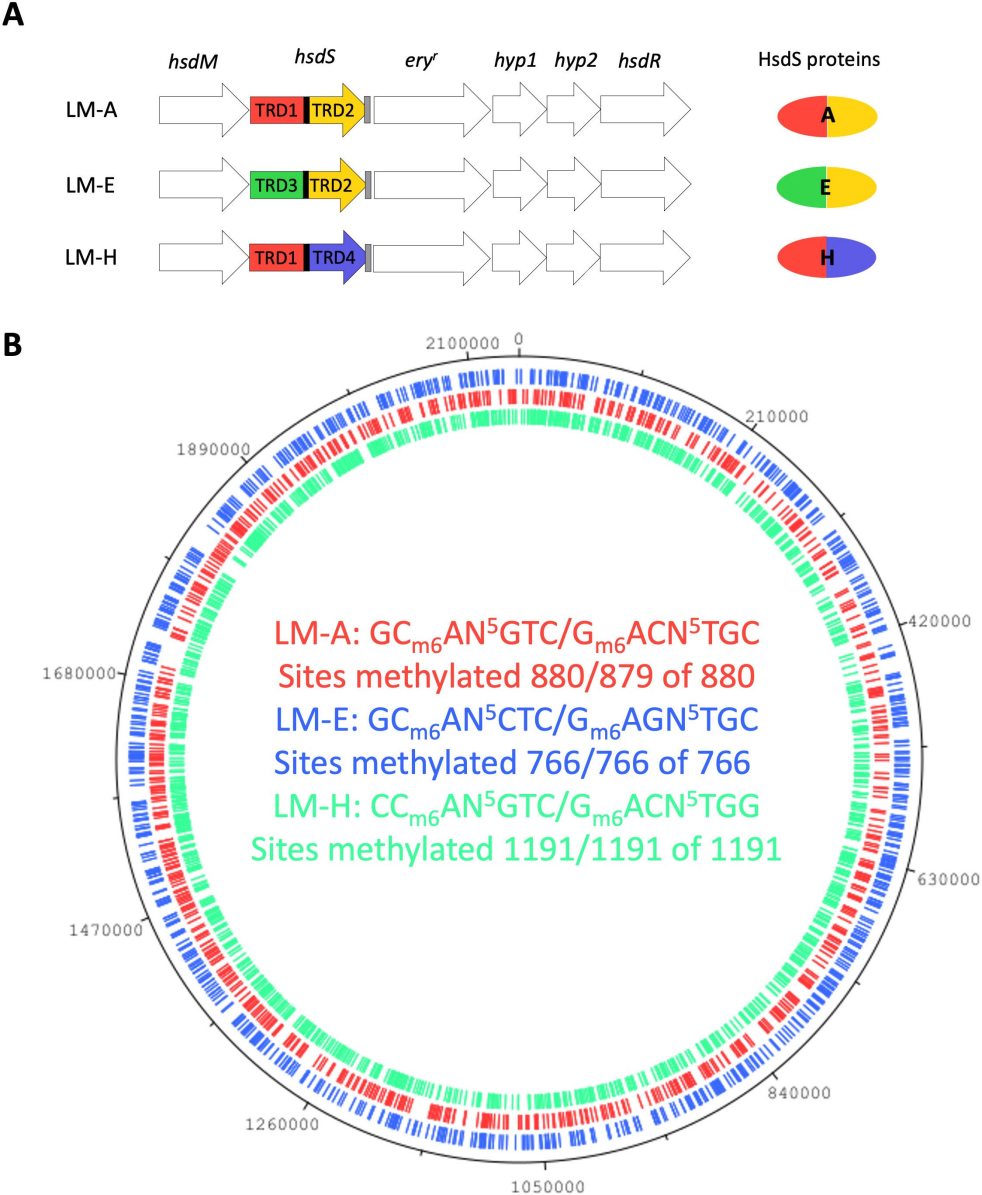
Locked mutants (LMs) have unique m6A methylation patterns. (A) Graphical representation of the SsuCC20p locus in the LMs. (B) The LM methylation patterns were plotted on the 861160 WT genome; m6A methylated sites in the genome are indicated on the genome with the color corresponding to the DNA motif.

To validate the predicted *hsdS* allele assignment to the observed methylation profiles in the WT 861160 strain, we generated locked mutants (LMs). In these mutants, the silent TRDs and *xerD* were replaced by an erythromycin resistance cassette, resulting in LMs expressing a single *hsdS* allele (Fig. 5A). The LMs expressed *hsdM*, *hsdS*, and *hsdR* (Fig. S4). SMRT and HiFi sequencing showed that each LM had a single unique m6A methylation profile (Fig. 5B) and validated the predicted *hsdS* allele assignment to the methylation profiles (Fig. 3C). Additionally, we identified the methylation profile of *hsdS* H (CC_m6_AN^5^GTC/G _m6_ACN^5^TGG). In contrast to the *hsdS* E methylation profile in WT, all potential methylation sites for *hsdS* E were methylated in LM-E. Of note, genome analysis showed that all the locked mutants had acquired an SNP in the *cps2F* gene (371A > G) compared to their parent strain. Similar to this shared SNP, the genomes of the LMs were identical differing only in their distinct *hsdS* alleles; therefore, we continued our study with these mutants.

### LMs have distinct transcriptomes and show differences in virulence in a zebrafish larvae infection model

**Fig 6:**
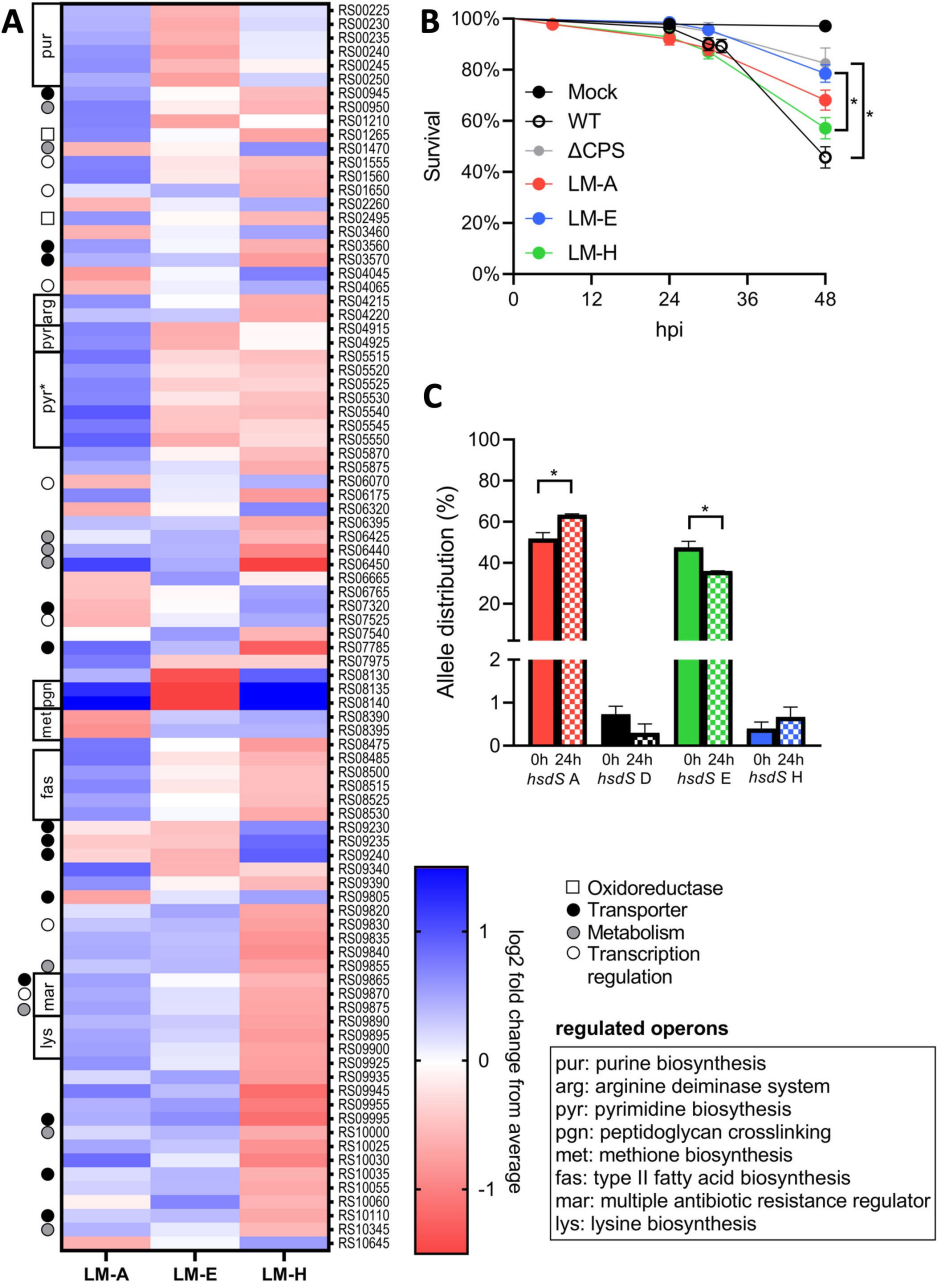
Locked mutants have distinct transcriptomes and virulence in a zebrafish larvae infection model. (A) Heatmap of the 90 differentially expressed genes (>2-fold, *P* < 0.05 ). Log2 fold change is expressed relative to the average of the three LMs. Scale endpoints are not absolute; genes with relative higher or lower expression have the endpoint color. pyr*, operon also contains genes predicted not to be involved in pyrimidine biosynthesis. For the RNA sequencing, two biological replicates for LM-A and three for LM-E and LM-H were used. (B) Zebrafish larvae (72 hours post fertilization) were infected via yolk sac injection with 2,700 CFU of *S. suis*, and survival was followed for 48 hours post infection (hpi). Per group, 20 larvae were infected in three to eight separate experiments. Data from individual experiments were pooled, and statistical difference was determined using a log-rank (Mantel-Cox) test with a Bonferroni correction for multiple testing. *P < 0.001. (C) hsdS allele distribution in WT 861160 at 0 hpi and 24 hpi in the zebrafish larvae. Allele abundance was computed by measuring the relative area under the curve of the different hsdS alleles using PeakScanner v3.0 in three biological replicates. Statistical differences was determined by a one-way analysis of variance with a Bonferroni correction for multiple testing. *P < 0.001.

Phase-dependent genome methylation profiles can result in distinct transcriptomes (13, 28), which can be dependent on growth conditions (28). To identify culture conditions that result in distinct transcriptomes between the LMs, we first quantified the expression levels of 24 genes in cultures grown in THY and in human serum. In an initial screening, the predicted promoter sequences of these genes contained at least one of the methylation profiles (for details, see supplemental materials). LMs showed differential gene expression exclusively when grown in serum (Fig. S5). Consequently, we chose serum as the culture condition for subsequent transcriptome analysis of the LMs. Samples clustered based on LM *hsdS* allele in a multidimensional scaling (MDS) plot (Fig. S6). A total of 90 genes were differentially expressed between the LMs (>2-fold between at least two LMs, *P* < 0.05), including multiple genes that are located within a single operon (Fig. 6A). Many differentially expressed genes are involved in bacterial growth, such as nutrient uptake, carbohydrate metabolism, fatty acid biosynthesis, purine biosynthesis, and pyrimidine biosynthesis (Fig. 6A; Table S5). Two genes (RS08135 and RS08140) named *murM* and *murN* that formed one operon and are predicted to encode amino acyl transferases that generate branched stem peptides in peptidoglycan (29) showed a 24- to 43-fold higher expression in LM-A and LM-H than in LM-E. None of the differentially expressed genes encoded known *S. suis* virulence factors.

The distinctive transcriptome of LMs could impact *S. suis* virulence (10, 13, 30). We evaluated the LMs’ virulence in a zebrafish larvae infection model, including WT and ΔCPS strains as controls. This model has been previously used to study the virulence of *S. suis* and other *Streptococcus* species (14–16, 19, 31). The genomic identical LMs (except for *hsdS* allele) showed differences in virulence with a reduced virulence for LM-E, resulting in a higher zebrafish survival than LM-H (Fig. 6B). LM-A showed an intermediate virulence that did not statistically significantly differ from LM-E or LM-H. The observed difference could not be attributed to difference in growth rate (Fig. S7A and B). Notably, the WT strain, still capable of phase variation, was the most virulent strain. Considering the LMs’ difference in virulence, we reasoned that the relative abundance of *hsdS* alleles in the WT strain could shift during zebrafish infection, as certain phases might contribute to bacterial survival or increased growth rate under these conditions. Quantification of the *hsdS* alleles at 0 h and 24 h indeed showed a relative increase of *hsdS* A and decrease in *hsdS* E (Fig. 6C). The relative increase of *hsdS* H did not reach statistical significance. Of note, *hsdS* D was detected for the first time.

## DISCUSSION

Epigenetic regulation of bacterial gene expression by phase-variable RM systems plays an important role in host-microbe interactions, including bacterial pathogenesis (7, 13, 28, 30, 32, 33). Here, we characterized the phase-variable type I RM system SsuCC20p that is encoded on a mobile element and is found in multiple streptococcal species including a zoonotic *S. suis* lineage. The SsuCC20p *hsdS* allele-dependent genome methylation impacted the *S. suis* transcriptome and virulence in a zebrafish larvae infection model.

To the best of our knowledge, in all phase-variable type I RM systems described so far, only the *hsdS* allele directly downstream of the *hsdM* is transcribed and involved in genome methylation (10, 13, 25, 26, 28, 30, 34). As a result, it was suggested that the other TRDs within the locus are merely present to function as DNA templates for recombination (34). Here, we demonstrated that additional *hsdS* alleles further downstream of *hsdM* are not always silent, since *hsdS* E of SsuCC20p is transcribed while downstream of *xerD* and not *hsdM*, and *hsdS E* is involved in methylation.

The observed TRD shuffling within SsuCC20p deviates from previously described TRD shuffling in phase-variable type I RM systems (10, 13, 25, 26, 28, 30, 34), as the observed TRD shuffling in SsuCC20p is a result of two concurrent TRD inversions or TRDs switching place (Fig. 1A). Unlike previously characterized phase-variable type I RM systems, which contain multiple unique sets of IRs, SsuCC20p has two identical sets of IRs (10, 13, 25, 26, 28, 30, 34). We hypothesize that the two sets of identical IRs, together with the site-specific recombinase encoded within SsuCC20p, allow for the observed TRD shuffling. Although we have established the indispensability of *xerD* for TRD shuffling, the precise molecular mechanism through which XerD facilitates these recombination events remains unknown. We speculate that the TRD shuffling within SsuCC20p results from coordinated double recombination or intramolecular recombination (35).

Our query for SsuCC20p in *S. suis* isolates identified four *hsdS* alleles, and three of these (A, E, and H) were initially detected in 861160 cultures grown overnight in THY. However, during quantification of the different *hsdS* alleles in the zebrafish infection, *hsdS* D could be detected albeit at a low abundance. The *in vitro* culture conditions influenced allele distribution such that the abundance of hsdS D was too low to be detected. In *S. pneumoniae*, the absence of specific *hsdS* alleles within an isolate under specific culture conditions was observed as well (13).

Most CC20 isolates including 861160 encode three type I RM systems (SsuCC20p, SsuPORF1588P, and SsuPORF1273P) (6), but only SsuCC20p was found to methylate the genome. In many prokaryotes, multiple (type I) RM systems methylate the genome simultaneously (10, 34, 36). Closer inspection of SsuPORF1588P showed that both *hsdS* have only a single TRD instead of the two TRDs that almost all functional type I RM *hsdS* encode (Fig. S8A) (8); thus, we speculate that SsuPORF1588P is not functional in methylation. SsuPORF1273P is phase variable (25), and the methylation profile of its two dominant alleles (A and B) has been solved by SMRT sequencing of *E. coli* overexpressing the *S. suis* methyltransferases, giving CC_m6_AN^8^CTT for allele A and CC_m6_AN^6^DNH (D = A/G/T, H = A/C/T, and *N* = A/C/G/T) for allele B (26). SsuPORF1273P expression when grown overnight on brain heart infusion (BHI) plate has been demonstrated, but genome methylation in *S. suis* has not been shown yet (25). In 861160, SsuPORF1273P appears functional and carries *hsdS* allele C (Fig. S8B); thus, SsuPORF1273P should be able to methylate the genome when expressed. The different culture condition (THY broth vs BHI plates [25]) could explain the absence of SsuPORF1273P methylation in our hands, although other mechanisms regulating transcription or translation of SsuPORF1273P could also be involved. The genome methylation by only a single type I RM system in the presence of multiple encoding RM systems, due to differences in transcription levels under specific culture conditions, has been observed in *Streptococcus pyogenes* as well (37).

The partial *hsdS* E and absent *hsdS* H methylation detected in 861160 WT are likely caused by the consensus approach used to extract the methylation profile from the SMRT sequencing reads, explaining why all potential methylation sites are methylated in LM-E and LM-H. A similar observation was made for the Spn556I in *S. pneumoniae* ST556 WT, in which all potential sites were methylated for the dominant *hsdS* allele but only a fraction, or none, of the potential sites were methylated for the minor *hsdS* alleles (10). Methylated nucleotides affect the interpulse duration measured during SMRT sequencing, which is used to identify methylation profiles (11). We used a consensus approach for methylation detection, in which SMRT reads are aligned to obtain the mean interpulse duration (IPD). We speculate that the IPD value of *hsdS* E or H methylated reads is averaged with the dominant *hsdS* A methylated reads to a level that the potential methylation sites are classified as unmethylated, leading to the partial *hsdS* E and absent *hsdS* H methylation profile detected in 861160 WT.

When grown in human serum, the unique methylation profiles of the LMs resulted in distinct transcriptomes. The differentially expressed genes are typically predicted to facilitate bacterial growth, such as the biosynthesis of nucleotides, amino acids, or type II fatty acids. Similar transcriptomic shifts have been observed in *S. suis* grown in cerebrospinal fluid (CSF) or blood, which indicate selective adaptations to these host niches (38). The distinct transcriptomes of SsuCC20p phases could potentially contribute to similar adaptions to host niches, thereby contributing to virulence. The genes *murM* and *murN* (RS08135 and RS08140) were most significantly differentially expressed between the LMs. MurM and MurN proteins synthesize branched stem peptides in the peptidoglycan layer (29). Although *murMN* were shown to affect β-lactam susceptibility (39), the LMs did not show differences in penicillin or ampicillin susceptibility (Table S6). *murMN* have been shown to affect the virulence of *S. pneumoniae* and *S. aureus*. In *S. pneumoniae*, *murMN* is essential for appropriate pneumolysin release (40), and *murM* deletion decreased the attachment of some LPXTG motif containing surface proteins to the bacterial surface (41). Transposon insertion mutants of *femA* and *femB*, *murMN* homologues in *S. aureus*, showed attenuated virulence in a murine bacteremia model (42). Similarly, in *S. suis*, decreased *murMN* expression could affect the appropriate release of suilysin or attachment of some LPXTG motif containing surface proteins. Therefore, we speculate that the reduced *murMN* expression in LM-E compared to LM-A and LM-H could have contributed to the reduced virulence of LM-E and decline of the *hsdS* E allele in the zebrafish larvae infection model.

The importance of epigenetic regulation by phase-variable RM systems has been demonstrated in many bacterial pathogens (7). Multiple lineages of *S. suis* carry phase-variable RM systems of which some are restricted to specific lineages (25). We characterized a phase-variable type I RM system in the newly identified zoonotic CC20 lineage. Pathogenicity in bacteria, including *S. suis* and the CC20 lineage, has been correlated with a reduction in gene content (5, 43, 44), including the loss of transcriptional regulators in virulent *S. suis* isolates (44). We propose that phase-variable RM systems offer transcriptional flexibility via epigenetic regulation, allowing a reduction in other transcriptional regulators in the genome while maintaining phenotypic diversity within the isolate that is needed to thrive or survive in new environments. Thus, explaining the co-occurrence of reduced gene content and phase-variable RM system acquisition in virulent and zoonotic *S. suis* lineages. Here, we characterized the phase-variable type I RM system SsuCC20p and demonstrated its impact on *S. suis* transcriptome and virulence via differential genome methylation.

## MATERIALS AND METHODS

### SsuCC20p identification in *S. suis*

A custom-made ABRicate (v1.0.1, https://github.com/tseemann/abricate) database containing the nucleotide sequences of the five genes and four TRDs present within the SsuCC20p locus (Table S1) was run (blastn) against a curated collection of 1,703 *S*. *suis* genome assemblies supplemented with 46 genome assemblies of recently sequenced European zoonotic *S. suis* isolates (45) (J. Brizuela, T. Roodsant, Q. Hasnoea, B. van der Putten, J. Kozakovac, H. C. Slotved, M. van der Linden, I. de Beer, E. Sadowyg, J. A. Saez-Nieto, V. Chalkeri, K. van der Ark, C. Schultsz, submitted for publication) using default settings. For the identification of SsuCC20p in other bacterial species, the ABRicate database was translated to protein and used to search with tblastn against the NCBI Refseq Genomes Bacterial Database, excluding *S. suis* using default settings. Hits within the same contig with a minimal identity of 90% for the non-phase-variable genes *hsdM*, *xerD*, and *hsdR* were selected for genomic region extraction. The extracted genomic regions containing SsuCC20p were aligned using easyfig (v2.2.5) (46).

### Bacterial strains and culture conditions

The *S. suis* strains used are listed in Table S4. *S. suis* was grown in THY at 37°C, supplemented with 200 µg/mL kanamycin, 2 µg/mL erythromycin, or 100 µg/mL spectinomycin when required.

### *hsdS* allele quantification

SsuCC20p *hsdS* allele quantification was adapted from previously published protocols for other phase-variable RM systems (13, 25). In short, FAM-labeled PCR fragments were amplified from genomic DNA using the primers 5′-6FAM-CTGGAGGGTGTTCTAATGATG-3′ and 5′-CCGCTCGCTATTTCCTA-3′. Fragments were gel purified and enzymatically digested with SmlI (NEB) for 1 h at 55°C. Digested fragments were diluted in HiDi formamide and ran on the ABI 3730XL DNA analyzer using the LIZ1200 size standard. *hsdS* alleles were quantified by measuring the area under the curve using Peak Scanner (v3.0).

### Cloning

Mutants listed in the Table S4 were created by homologous recombination using peptide-induced competence and mutator fragments containing an antimicrobial resistance marker (kan^r^ [47], ery^r^ [48], spc^r^ [49]) with >500-bp homologous flanking regions as previously published (50). Briefly, 5 µM of the ComS13-21 competence inducing peptide and 1–5 µg of mutator fragment were added to *S. suis* grown to an optical density (OD, 600 nm) of 0.05 and incubated for 2 h at 37°C. Hereafter, transformants were selected on THY agar plates containing corresponding antibiotics. Mutator fragments were generated using overlapping PCR or by PCR amplification of a synthesized gblock (IDT DNA technologies) for the Δ*hsdS* mutant, with the primers listed in the Table S5. Mutants were checked by PCR and sequencing.

### SMRT sequencing and methylome analysis

An overnight culture of *S. suis* was diluted 500× in fresh pre-warmed THY and incubated at 37°C until it reached an OD of 0.30–0.45. Genomic DNA was isolated using the Wizard genomic DNA purification kit (Promega) according to manufacturer’s protocol. DNA was quantified with Qubit, and DNA integrity was assessed on a 0.7% agarose gel. For detailed sequencing methods, see supplemental materials. Briefly, sequencing libraries were constructed from sheared genomic DNA (5–20 kb) and sequenced on a SMRT Cell 8M using a PacBio sequel II(e) system (Pacific Biosciences).

### Oxford Nanopore Technologies sequencing

Genomic DNA of a *S. suis* culture grown overnight in THY was isolated using the MagAttract high-molecular-weight DNA extraction kit (Qiagen). DNA was quantified with Qubit, and DNA integrity was assessed on a 0.7% agarose gel. Library preparations and Nanopore sequencing were performed as previously described (24).

### Serum collection

Blood was collected from healthy volunteer donors after informed consent into CAT serum clot activator tubes (Greiner Bio-One). After 30 min, serum was separated from clotted blood by centrifugation for 15 min at 2200 × *g*. Serum of four donors was pooled and stored at −70°C.

### Transcriptome analysis

RNA isolation, cDNA synthesis, and qPCR are described in the supplemental materials. For RNA sequencing, RNA isolation and sequencing were performed on three independent biological replicates; the RNA isolation protocol was adapted from reference 51. Briefly, overnight cultures were washed once with PBS and diluted to an OD of 0.5; then, cultures were inoculated 1:10 in pooled human serum. Cultures were pelleted in (mid) exponential phase and washed once with PBS before resuspension in 1.8-mL RNAlater (Invitrogen AM7020) and stored at 4°C. After 24 h, samples were moved to −70°C. RNA isolation and sequencing were performed by GenomeScan (The Netherlands). Briefly, RNA was isolated with the MagNAPure96 using the Viral RNA small volume kit (Roche 06543588001). After DNase treatment (NEB M0303), rRNA depletion and fragmentation (fast select 5S/16S/23S, Qiagen, 335921), libraries were constructed using the NEBNext Ultra II Directional RNA Library Prep Kit for Illumina (NEB E7760S/L). Libraries were sequenced on a Novaseq 6000 (Illumina) using the Illumina data analysis pipeline RTA3.4.4 and BclConvert v3.10.5 yielding ±10 million reads per sample.

Reads were trimmed with Trimmomatic (52) and aligned to the 861160 genome (24) using Bowtie2 (53), from which counts per gene were generated using htseq-count (54), with the SsuCC20p locus excluded from counting. One of the three biological replicates of LM-A was excluded due to contamination with human reads. Differential expression analyses was performed in Degust (v4.1.1) (55) using voom/limma (56, 57) after filtering out lowly expressed genes (minimal count per million 1.45 in at least two samples). Degust input data can be found in Table S7.

### Zebrafish larvae infection

Zebrafish larvae infections were performed as previously published (15) with minor adjustments. *S. suis* glycerol stocks were prepared to ensure consistent inocula in between experiments. *S. suis* cultures grown to an OD of 0.4 were frozen in 15% glycerol and stored at −70°C. For each experiment, stocks were thawed and washed once with PBS before resuspension in injection buffer (0.125% phenol red; Sigma P0290 in PBS) to obtain a bacterial suspension of 2,700 ± 300 CFU/nL. Bacterial inoculum was quantified each infection experiment by serial dilution and plating on blood agar plates.

Adult zebrafish were handled in compliance with the local animal welfare regulations approved by the local animal welfare committee (DEC) and were maintained according to standard protocols (www.zfin.org). Experiments with zebrafish embryos younger than 5 days post fertilization did not need ethical permission. Eggs were harvested within 1 h after spawning and kept in E3 medium (www.zfin.org) at 28°C before further use. At 72 h post fertilization, vital zebrafish larvae were anesthetized with 0.4% Tricaine in H_2_O and injected in the yolk sac with 1 nL of inoculum or injection buffer (mock). Injected larvae were kept grouped (20 per petri dish) in E3 medium at 28°C. Zebrafish survival was assessed by visual assessment of a heartbeat at 6, 24, 30, and 48 hpi, and diseased zebrafish larvae were removed.

To quantify the abundances of the SsuCC20p alleles, 10 viable zebrafish larvae were collected at 0 and 24 hpi. Zebrafish larvae were washed once with 50 mM EDTA and subsequently disrupted by bead beating (60 s, 4,000 speed); the obtained sample was stored at −20°C. Genomic DNA was isolated from the mixture using the Wizard genomic DNA purification kit (Promega) according to manufacturer’s protocol, and *hsdS* allele abundances were quantified as mentioned earlier.

### Statistical analysis

All statical analyses were performed using GraphPad Prism (v9.3.1), and statistical test and *P* values are indicated in figure legends.

## Data Availability

Raw (DNA/RNA) sequencing data and genome assemblies have been deposited in ENA under the accession numbers listed in the Table S4 and Table S8.

## SUPPLEMENTAL MATERIAL

Fig. S1 mbio.02259-23-s0001.tif Only SsuCC20p conformations with a single complete hsdS can be detected in 861160 gDNA by PCR.

Fig. S2 mbio.02259-23-s0002.tif Methylome of WT 861160.

Fig. S3 mbio.02259-23-s0003.tif *In silico* assignment of hsdS allele to target DNA motif.

Fig. S4 mbio.02259-23-s0004.tif Verification of expression of hsdM, hsdS, and hsdR in locked mutants.

Fig. S5 mbio.02259-23-s0005.tif Locked mutants of SsuCC20p show differential gene expression exclusively when grown on serum.

Fig. S6 mbio.02259-23-s0006.tif MDS plot of RNAseq data obtained from the LMs.

Fig. S7 mbio.02259-23-s0007.tif Locked mutant growth rate.

Fig. S8 mbio.02259-23-s0008.tif SsuPORF1588P and SsuPORF1273P bioinformatic locus analysis.

Supplemental methods mbio.02259-23-s0009.docx Details of SMRT sequencing and methylome analysis, identification of potentially differentially expressed genes in LMs, RNA isolation and RT-(q)PCR, and antimicrobial susceptibility testing.

Supplemental tables mbio.02259-23-s0010.xlsx Tables S1 to S9.

ASM does not own the copyrights to Supplemental Material that may be linked to, or accessed through, an article. The authors have granted ASM a non-exclusive, world-wide license to publish the Supplemental Material files. Please contact the corresponding author directly for reuse.
