# Supplementary figures and images for: The streptococcal phase-variable type I restriction modification system SsuCC20p dictates the methylome of Streptococcus suis impacting the transcriptome and virulence in a zebrafish larvae infection model
Source: mBio. 2023 Dec 8;15(1):e02259-23. doi: 10.1128/mbio.02259-23 (PMC10790761; doi:10.1128/mbio.02259-23)

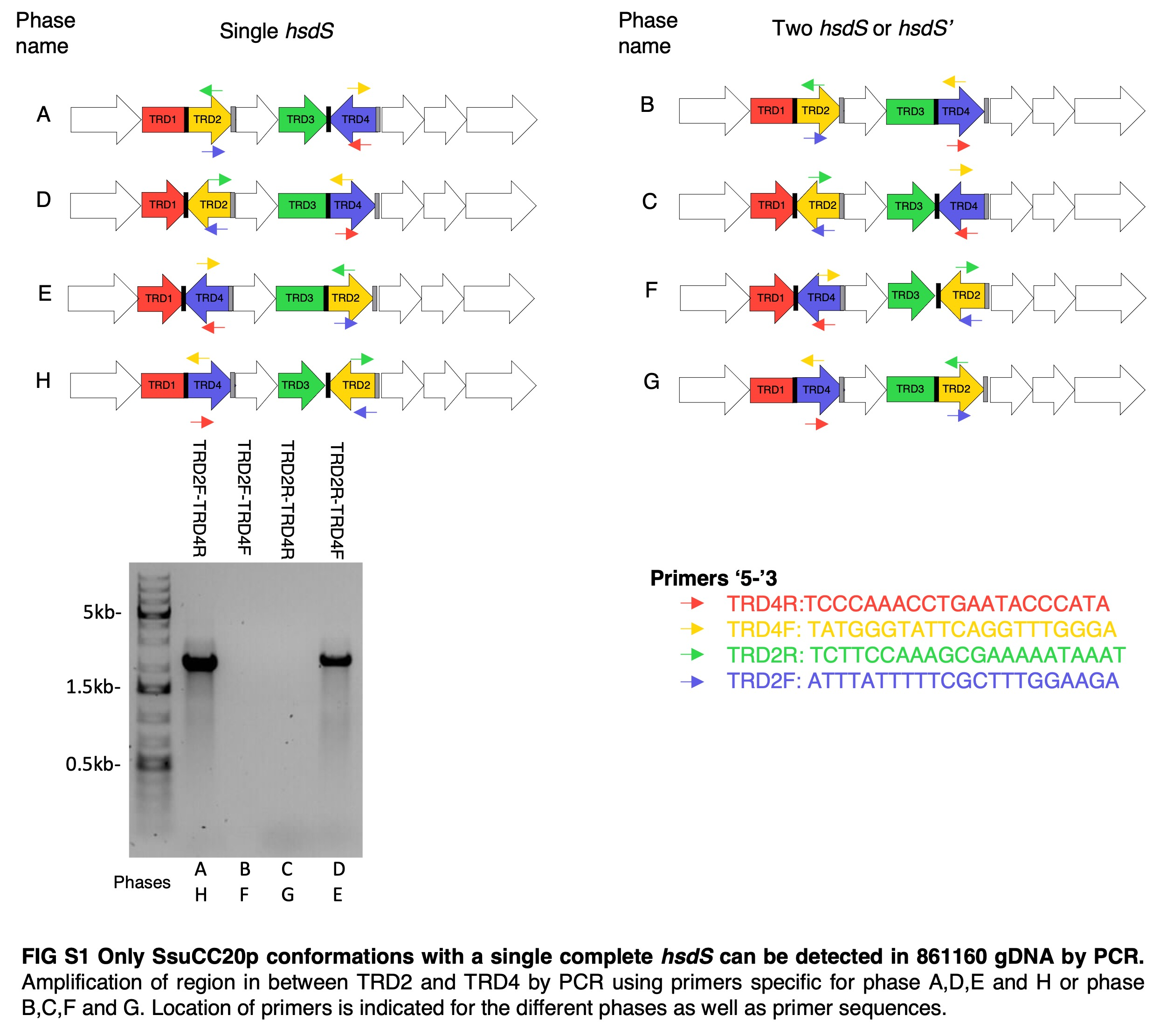

Supplement: Fig. S1 — Only SsuCC20p conformations with a single complete hsdS can be detected in 861160 gDNA by PCR. [file mbio.02259-23-s0001.tif]

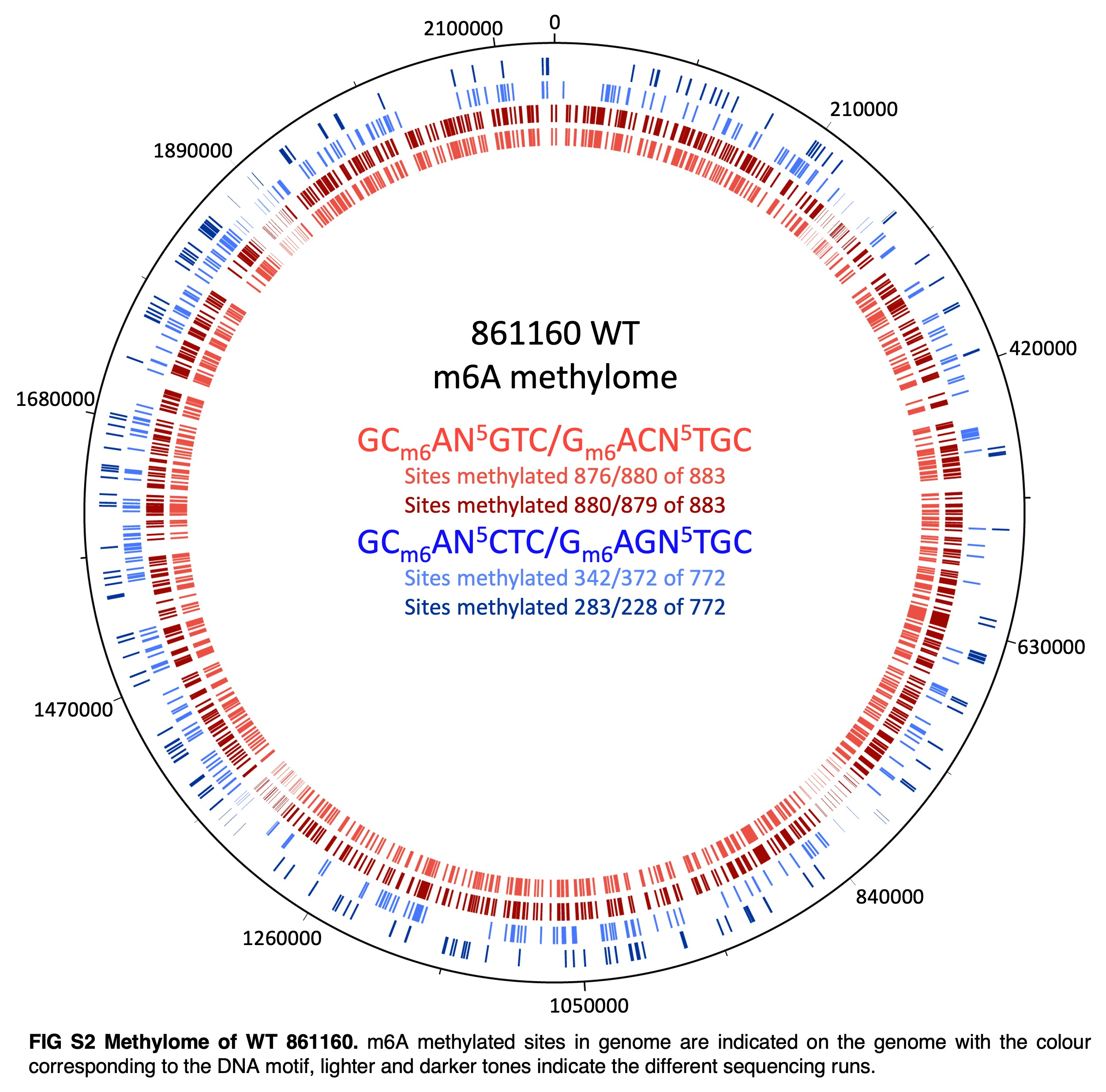

Supplement: Fig. S2 — Methylome of WT 861160. [file mbio.02259-23-s0002.tif]

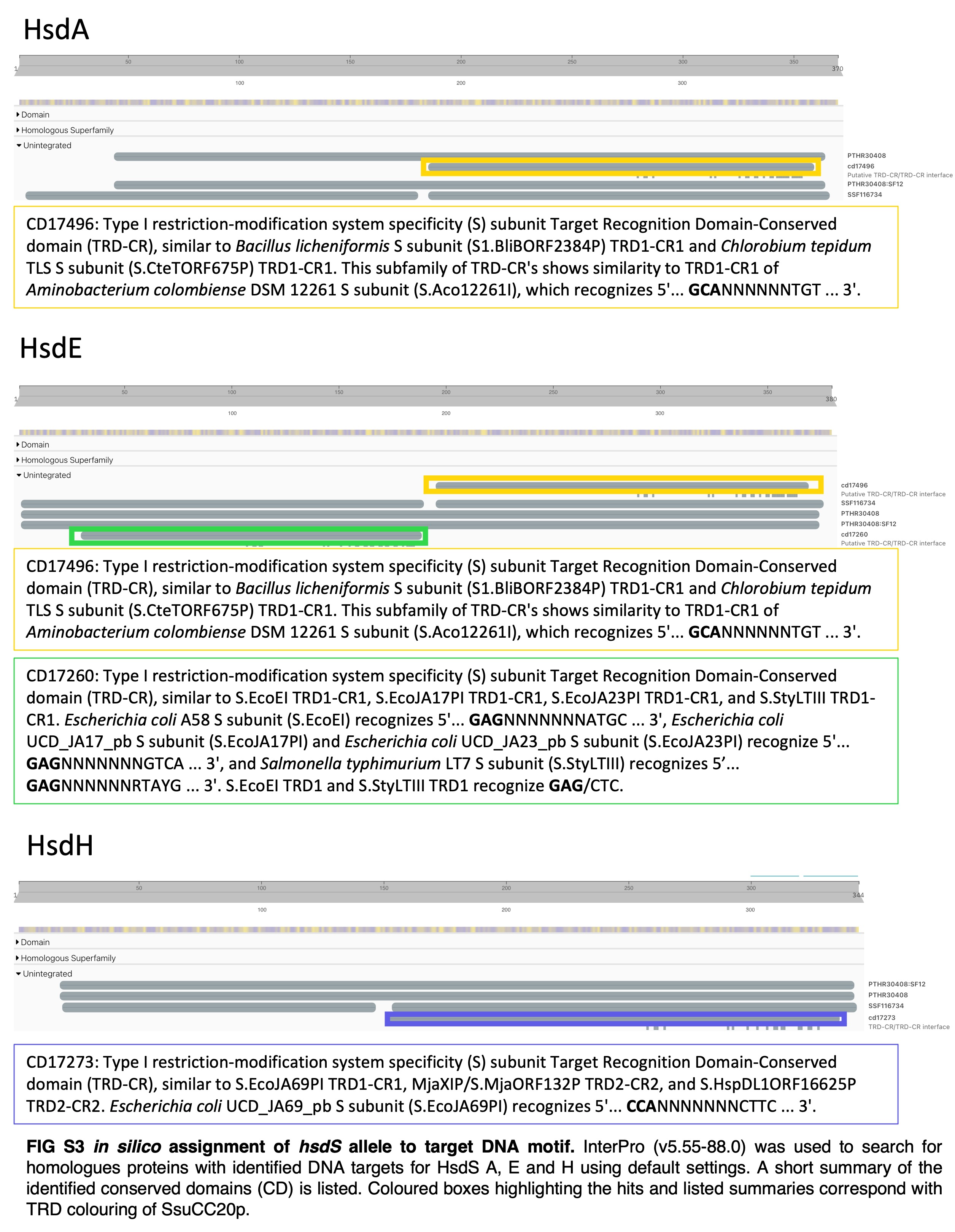

Supplement: Fig. S3 — In silico assignment of hsdS allele to target DNA motif. [file mbio.02259-23-s0003.tif]

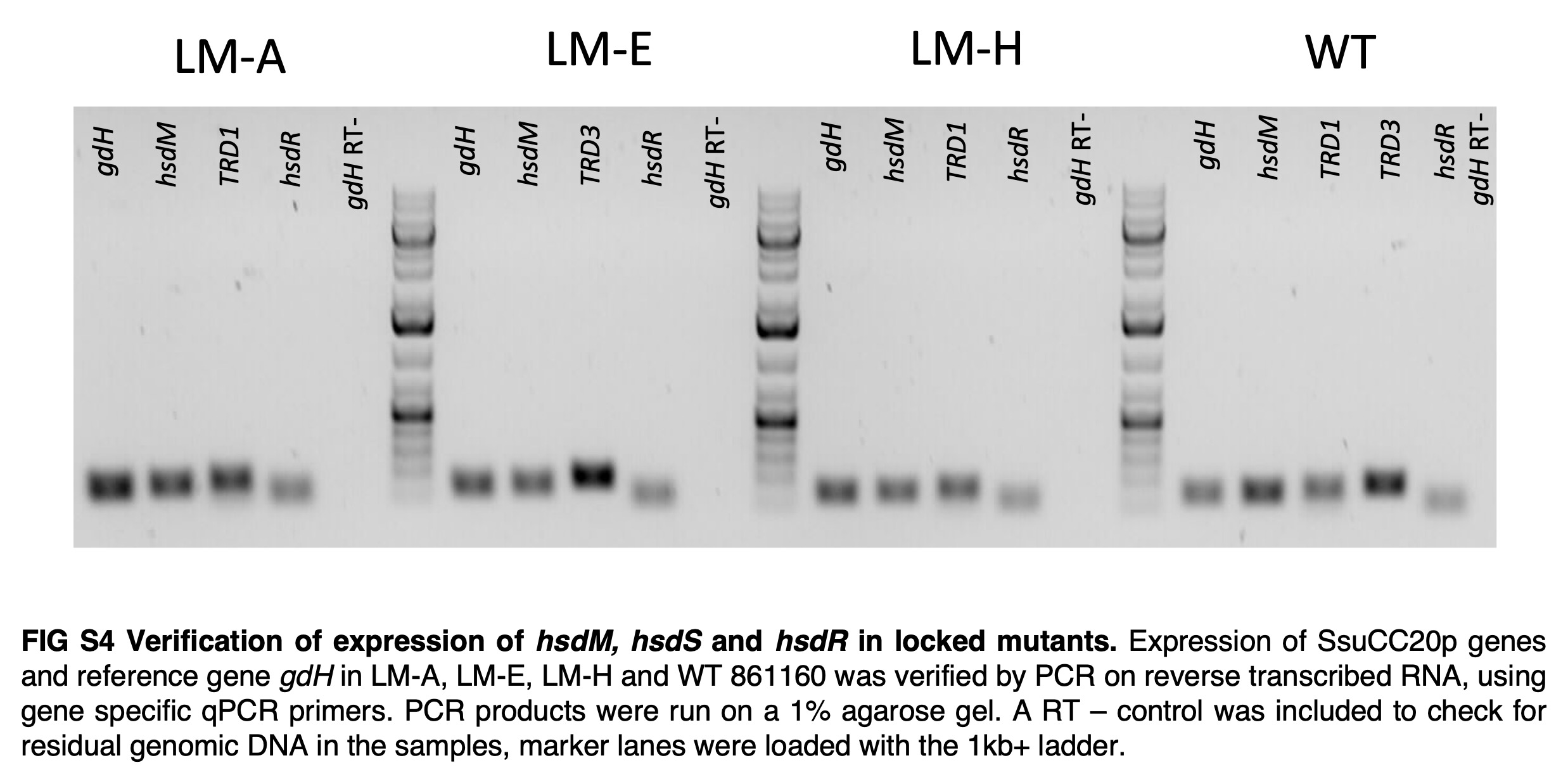

Supplement: Fig. S4 — Verification of expression of hsdM, hsdS, and hsdR in locked mutants. [file mbio.02259-23-s0004.tif]

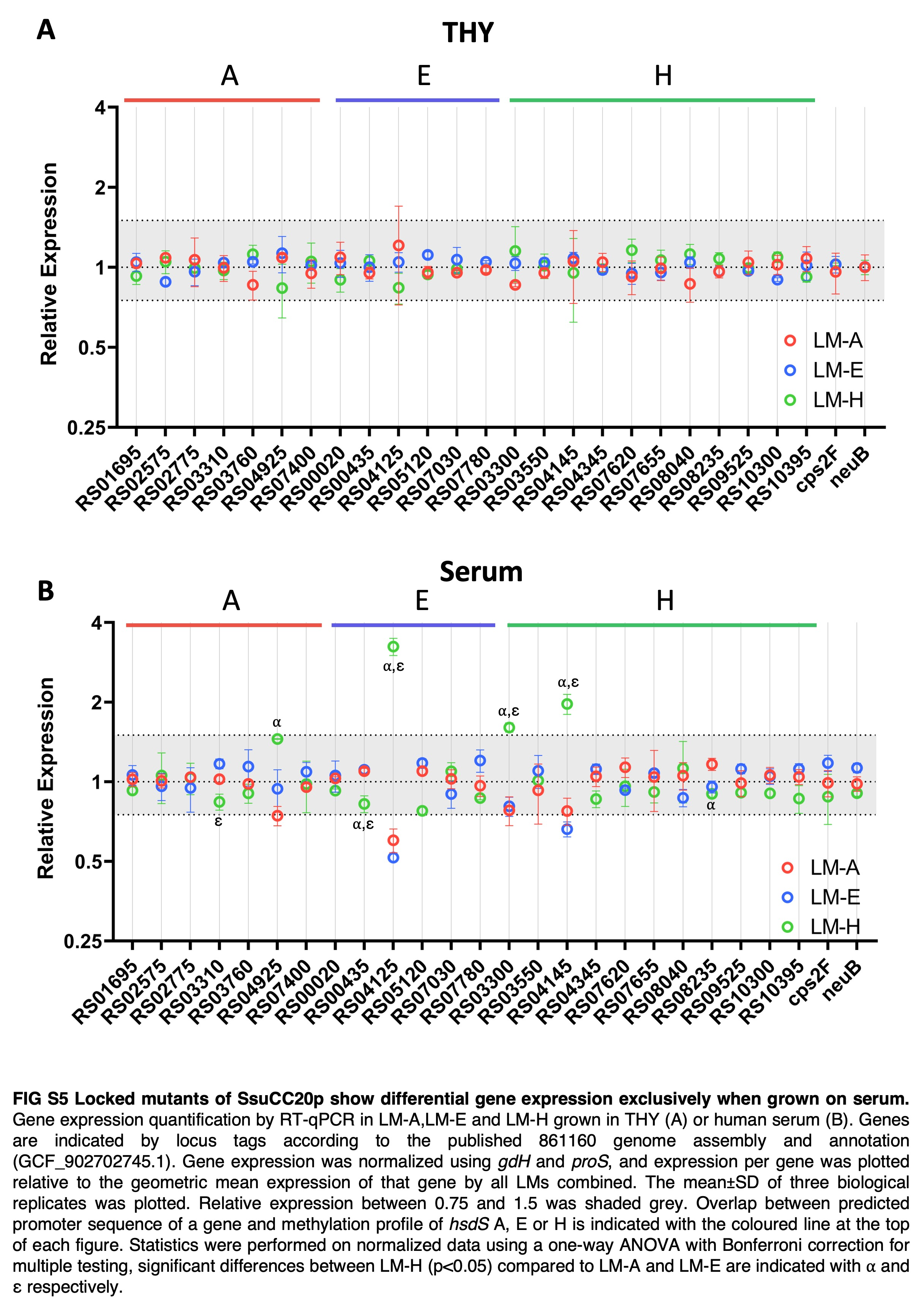

Supplement: Fig. S5 — Locked mutants of SsuCC20p show differential gene expression exclusively when grown on serum. [file mbio.02259-23-s0005.tif]

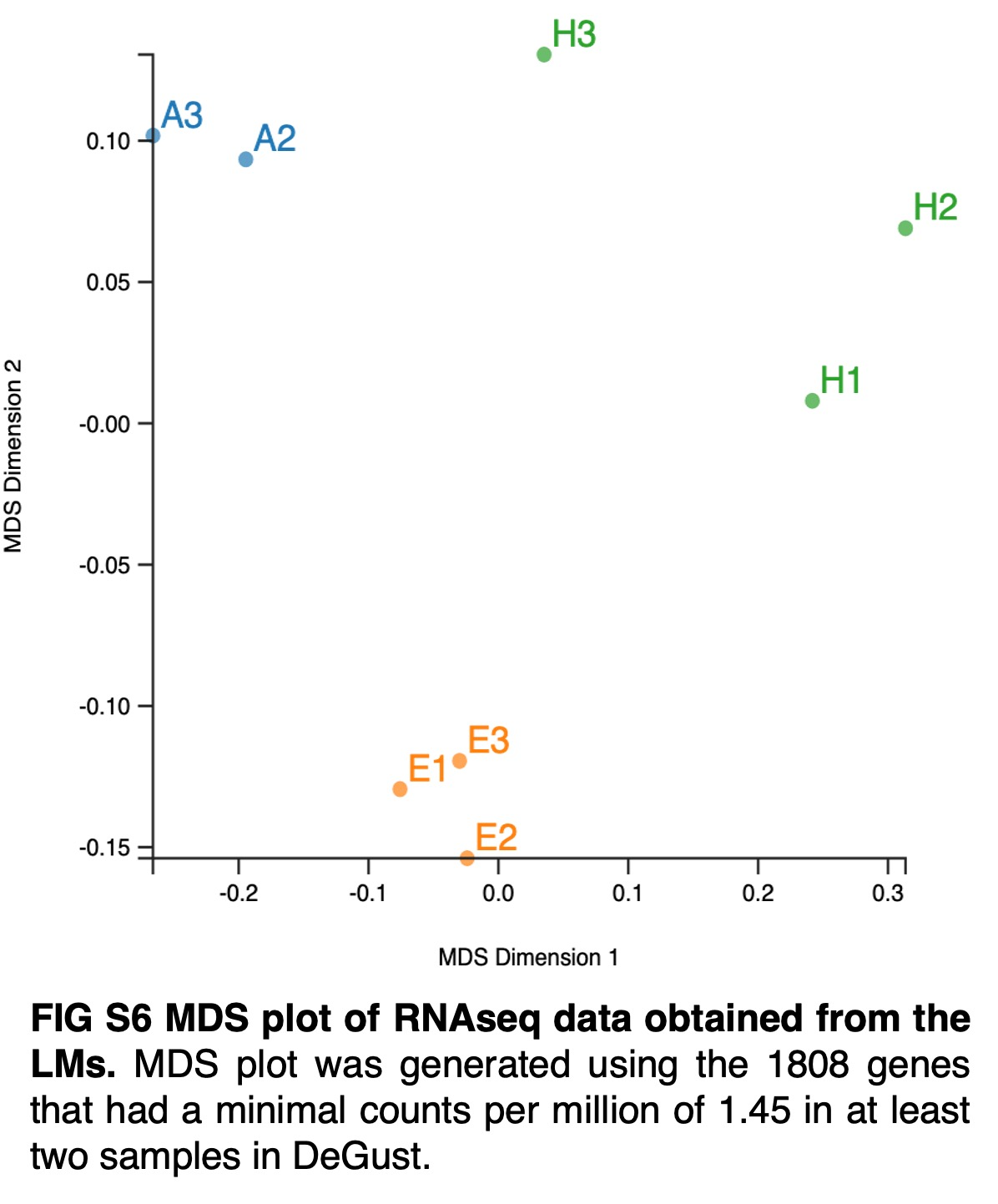

Supplement: Fig. S6 — MDS plot of RNAseq data obtained from the LMs. [file mbio.02259-23-s0006.tif]

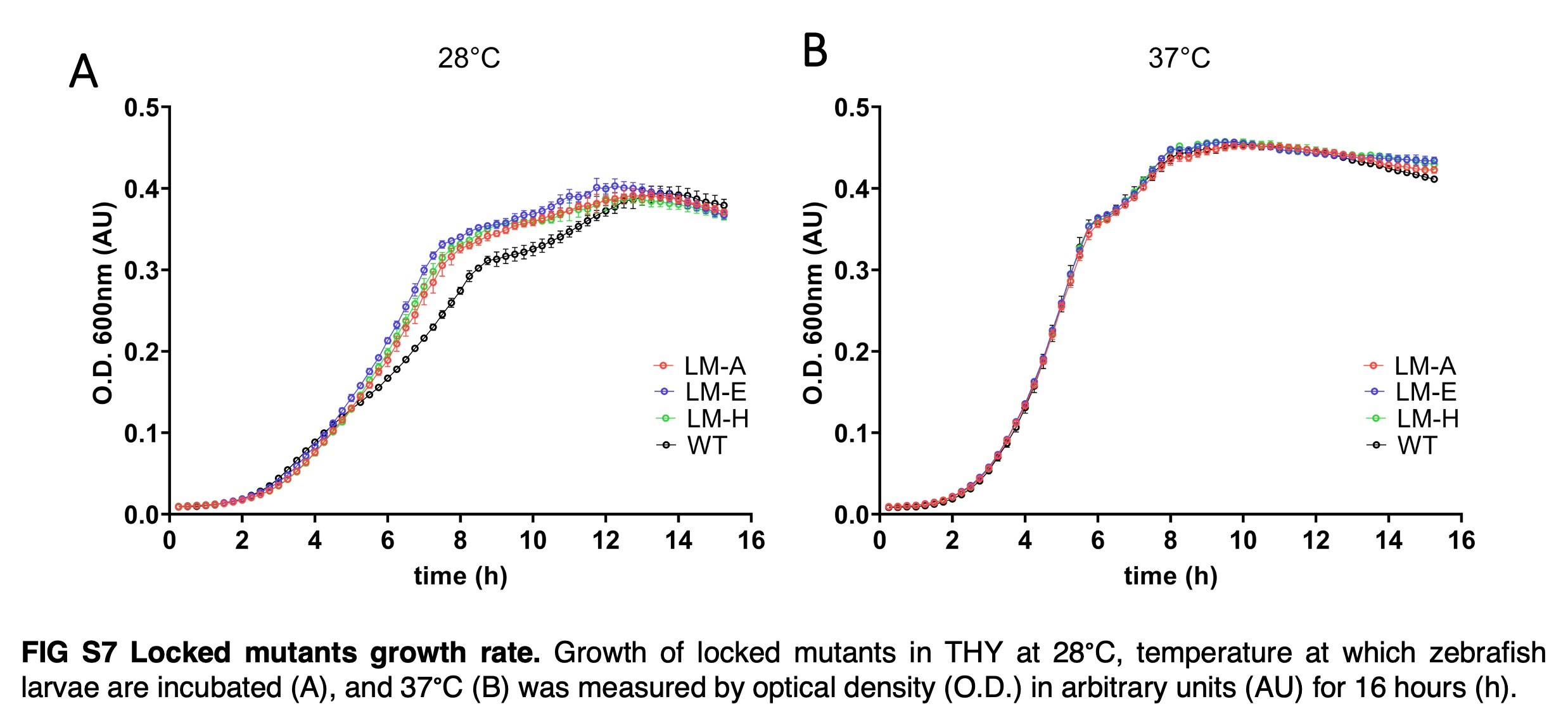

Supplement: Fig. S7 — Locked mutant growth rate. [file mbio.02259-23-s0007.tif]

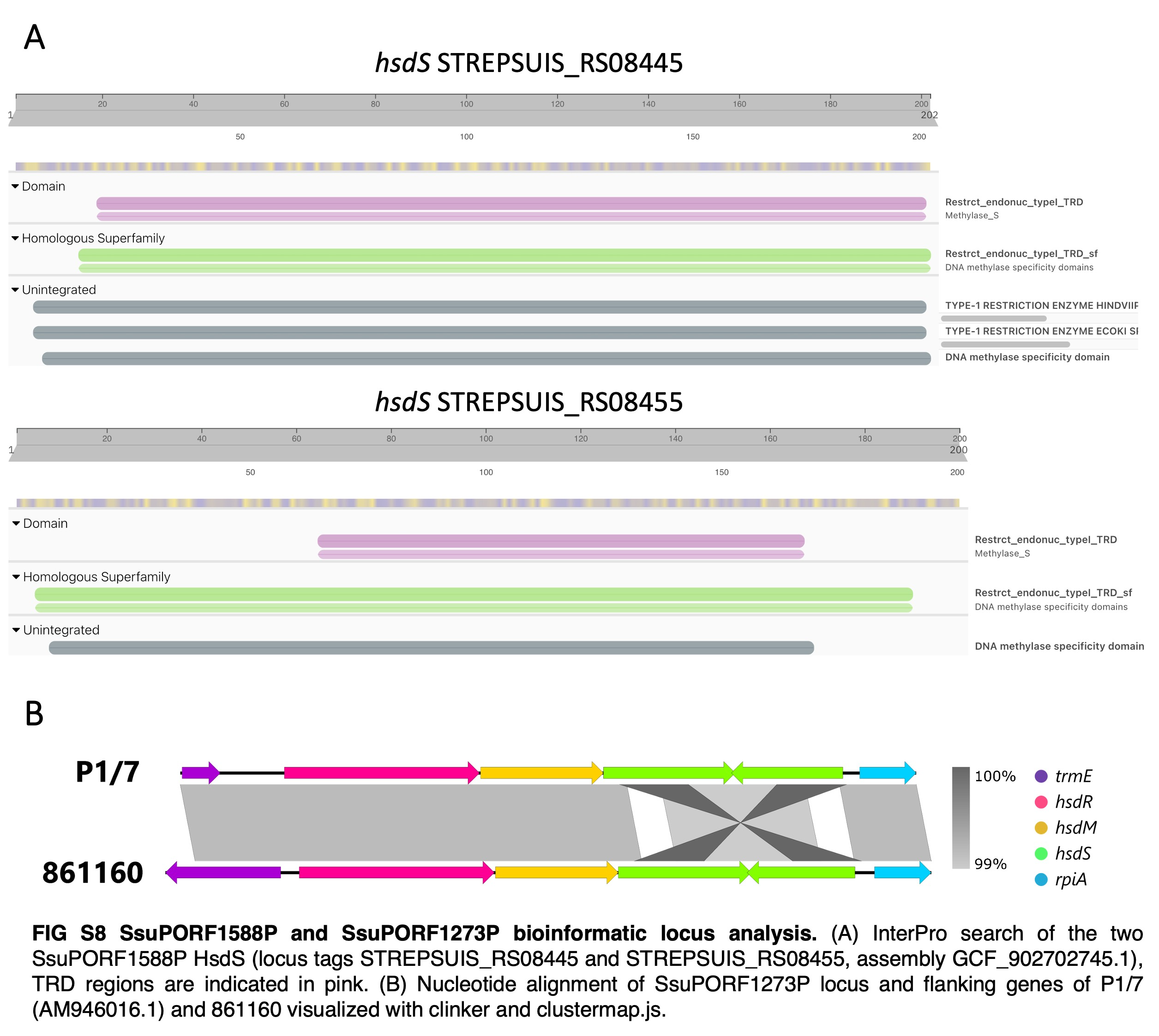

Supplement: Fig. S8 — SsuPORF1588P and SsuPORF1273P bioinformatic locus analysis. [file mbio.02259-23-s0008.tif]
