# Supplementary material for: The streptococcal phase-variable type I restriction modification system SsuCC20p dictates the methylome of Streptococcus suis impacting the transcriptome and virulence in a zebrafish larvae infection model
Source: mBio. 2023 Dec 8;15(1):e02259-23. doi: 10.1128/mbio.02259-23 (PMC10790761; doi:10.1128/mbio.02259-23)
Supplement: Supplemental methods — Details of SMRT sequencing and methylome analysis, identification of potentially differentially expressed genes in LMs, RNA isolation and RT-(q)PCR, and antimicrobial susceptibility testing. [file mbio.02259-23-s0009.docx]

**Supplemental materials**

**Details SMRT sequencing and methylome analysis**

For sequencing at the Radboud University Medical Centre (Radboud University), integrity was also assessed on a TapeStation 4150 system (Agilent) using a Genomic DNA Screentape. The multiplexed library was constructed using the SMRTbell® Express Template Prep kit 2.0 (Version 08, November 2021) according to manufacturer’s protocol, except for the DNA shearing. Genomic DNA samples were sheared to ±20 kb using The Megaruptor® 3 at speed 32 (100 μl of 36 ng/μl per sample). The pooled samples were purified using 0.45X AMPure PB beads and sequenced on a SMRT Cell 8M using a PacBio Sequel IIe system (Pacific Biosciences).

Sequencing at SNPSaurus (University of Oregon) was performed as previously described (1). Briefly, SMRTbell template sequencing libraries were prepared using the Sequel II binding kit 2.0 from genomic DNA that was sheared to an average length of ± 5-10 kb using g-TUBEs (Covaris). End repaired DNA was ligated to hairpin adapters and incomplete SMRTbell templates were degraded with exonuclease III (NEB) and exonuclease VII (USB). After primer annealing, samples were sequenced on a SMRT Cell 8M using a PacBio sequel II system (Pacific Biosciences) using standard protocols for long insert libraries.

The complete genome of 861160 was generated using PacBio HiFi CCS reads assembled using Flye v2.9 with the “--pacbio-hifi” setting. PacBio subreads were subsequently mapped onto the constructed reference genome using pbmm2 v1.4.0. ipdSummary (SMRTtools v11.0.0) was used to search for specifically m6A and m4C methylated sites. Methylated motifs were defined based on ipdSummary output using the "find” subcommand of motifMaker (SMRTtools v11.0.0), after which the reprocess subcommand of motifMaker was used to link specific methylation sites to their motifs. Default settings were used unless stated otherwise.

**Identification of potentially differentially expressed genes in LMs**

The 861160 genome was manually screened for genes that had either of the three methylation profiles within 100 bp upstream of their start codon in Artemis (2, 3), and the 81 bp directly upstream were extracted. Promoter sequences were identified within these 81 bp with iPro70-FMWin (4), BRPOM (5), iPromoter-2L(6), promotech (7) and previously identified *S. suis* promoters (8), using default settings. Genes downstream of predicted promoter sequences (based on BPROM or Promotech) that had overlap with either of the three methylation profiles were selected for RT-qPCR.

**RNA isolation and RT-(q)PCR**

For growth in serum the protocol was adapted from (9), an overnight culture was diluted to an OD of 0.4 and diluted 10x in serum, then grown for 150 min at 37 °C at 5% CO_2_. For growth in THY, an overnight culture of *S. suis* was diluted 500x in fresh pre-warmed THY and incubated at 37 °C until it reached OD of 0.30-0.45. Culture (10 mL) was pelleted (10 min, 4000 xg, 4**°**C) and resuspended in 1.0 mL Trizol (Invitrogen). Cells were lysed by beat-beating in a MagnaLyser (30 s, 6000 speed) using 0.1 mL zirconium sand and 3x2mm glass beads, with one round of beat-beating for THY cultures and five round for serum cultures. RNA was purified from lysed cells using the direct-zol RNA miniprep kit (Zymo Research). Genomic DNA was removed with the TURBO DNA free kit (Invitrogen) in presence of SUPERase RNAse inhibitor (Invitrogen). cDNA was made from RNA (2.0 μg for THY and 0.6 μg for serum) with Superscript IV first strand synthesis kit (Invitrogen) using random hexamers. Gene expression was assessed by qPCR using primers listed in the supplemental material Table S9 and the GO Taq PCR kit (Promega). Gene expression was quantified using SYBR Green (Roche) in a CFX96 or CFX384 (BioRad) and analyzed using LinRegPCR (10, 11), using *proS* and *gdH* as reference genes (12, 13). All kits were used according to manufacturer’s protocol.

**Growth Curve**

Overnight cultures were diluted to an OD of 0.02 in fresh THY broth and 150 µL was distributed in a 96 well flat bottom plate. Bacterial growth at 37 °C or 28 °C without shaking was monitored by measuring the OD every 10 min for 16h in Biotek Synergy H1 plate reader. The assay was performed with three biological replicates.

**Antimicrobial susceptibility testing**

Disc diffusion assays (BD) were performed as described by the EUCAST guidelines. In brief, strains were grown overnight on blood agar plates, and their initial concentration adjusted using MacFarland standards. Bacterial lawns were incubated with the appropriate antimicrobial discs.

**References**

1. Tram G, Jen FE-C, Phillips ZN, Timms J, Husna A-U, Jennings MP, Blackall PJ, Atack JM. 2021. Streptococcus suis Encodes Multiple Allelic Variants of a Phase-Variable Type III DNA Methyltransferase, ModS, That Control Distinct Phasevarions. mSphere 6:e00069-21.

2. Carver T, Harris SR, Berriman M, Parkhill J, McQuillan JA. 2012. Artemis: An integrated platform for visualization and analysis of high-throughput sequence-based experimental data. Bioinformatics 28:464–469.

3. van der Putten BCL, Roodsant TJ, Haagmans MA, Schultsz C, van der Ark KCH. 2020. Five Complete Genome Sequences Spanning the Dutch Streptococcus suis Serotype 2 and Serotype 9 Populations. Microbiol Resour Announc 9.

4. Rahman MS, Aktar U, Jani MR, Shatabda S. 2019. iPro70-FMWin: identifying Sigma70 promoters using multiple windowing and minimal features. Mol Genet Genomics 294:69–84.

5. Solovyev V, Salamov A. 2011. Automatic annotation of microbial genomes and metagenomic sequences. Metagenomics its Appl Agric Biomed Environ Stud 62–78.

6. Liu B, Yang F, Huang DS, Chou KC. 2018. IPromoter-2L: A two-layer predictor for identifying promoters and their types by multi-window-based PseKNC. Bioinformatics 34:33–40.

7. Chevez-Guardado R, Peña-Castillo L. 2021. Promotech: a general tool for bacterial promoter recognition. Genome Biol 22:1–16.

8. Wu Z, Wu C, Shao J, Zhu Z, Wang W, Zhang W, Tang M, Pei N, Fan H, Li J, Yao H, Gu H, Xu X, Lu C. 2014. The Streptococcus suis transcriptional landscape reveals adaptation mechanisms in pig blood and cerebrospinal fluid. Rna2014/04/25. 20:882–898.

9. Fredriksen S, Ruijten SDE, Murray GGR, Juanpere-Borràs M, van Baarlen P, Boekhorst J, Wells JM. 2023. Transcriptomics in serum and culture medium reveal shared and differential gene regulation in pathogenic and commensal Streptococcus suis. Microb genomics 9.

10. Ruijter JM, Ramakers C, Hoogaars WMH, Karlen Y, Bakker O, van den hoff MJB, Moorman AFM. 2009. Amplification efficiency: Linking baseline and bias in the analysis of quantitative PCR data. Nucleic Acids Res 37:e45–e45.

11. Ramakers C, Ruijter JM, Lekanne Deprez RH, Moorman AFM. 2003. Assumption-free analysis of quantitative real-time polymerase chain reaction (PCR) data. Neurosci Lett 339:62–66.

12. Ferrando ML, Willemse N, Zaccaria E, Pannekoek Y, Van Der Ende A, Schultsz C. 2017. Streptococcal Adhesin P (SadP) contributes to Streptococcus suis adhesion to the human intestinal epithelium. PLoS One2017/04/14. 12:e0175639.

13. Ferrando ML, Van Baarlen P, Orrù G, Piga R, Bongers RS, Wels M, De Greeff A, Smith HE, Wells JM. 2014. Carbohydrate availability regulates virulence gene expression in Streptococcus suis. PLoS One 9:e89334.
